# Community-based participatory research to guide adoption of culturally responsive trauma-informed HIV care throughout Nashville, Tennessee

**DOI:** 10.21203/rs.3.rs-3739954/v1

**Published:** 2023-12-15

**Authors:** Lauren Brown, Jessica Perkins, Jessica Acuña, Julie Thacker, Clare Bolds, Mary Hawkins, Jamie Stewart, Julie Barroso, Sadie Sommer, Joshua Van der Eerden, Bryan Heckman, Amna Osman, Tarik Smith, LaToya Alexander, Allie Harvick, Tiye Link, Anita Crawley, Rosemary Nabaweesi, Maria Aboubaker, Joanna Shaw-KaiKai, Norman Foster, Beverly Glaze-Johnson, Jessica Hoke, Carolyn Audet, Jessica Sales, April Pettit

**Affiliations:** Meharry Medical College; Vanderbilt University; Meharry Medical College; Virginia Health Department; Vanderbilt University Medical Center; Nashville CARES; Meharry Medical College; Vanderbilt University; Vanderbilt University; Vanderbilt University; Fox Foundation; Nashville CARES; Meharry Medical College; Meharry Medical College; Vanderbilt University Medical Center; Nashville CARES; Street Works; Meharry Medical College; Nashville CARES; Metropolitan Public Heath Department Nashville; Metropolitan Public Heath Department Nashville; Metropolitan Public Heath Department Nashville; Nashville CARES; Vanderbilt University; Emory University; Vanderbilt University Medical Center

## Abstract

**Background:**

Psychological trauma is a highly prevalent driver of poor health among people with HIV (PWH) in the Southern United States (U.S.). Trauma-informed care (TIC) has potential to advance national Ending the HIV Epidemic (EHE) goals, but formative research is needed to tailor TIC implementation to complex and interdependent HIV networks. Methods: We applied a community-based participatory research (CBPR) approach to iteratively engage personnel from high volume HIV care institutions in Nashville, Tennessee. Current practices and potential implementation determinants were identified through participatory process mapping (PM) and key informant interviews. The Consolidated Framework for Implementation Research (CFIR) was applied to deductively code interview data. Personnel attending a dissemination summit developed a network-wide implementation plan.

**Results:**

Data were collected with personnel from five institutions (e.g., community-based organizations, primary care clinics, public health department), for PM (n=48), interviews (n=35), and the summit (n=17). Results suggest there are limited trauma screenings, assessments, and services across the network. Relevant Characteristics of Individuals included a trauma-sensitive workforce committed to continuous learning and TIC adoption. Relevant Inner Setting Factors were networks and communications, with strong tension for change, high compatibility with TIC, and need for advancing cultural responsiveness. Relevant Outer Setting Factors included patient needs and resources and cosmopolitanism, with need for better leveraged mental health services. Relevant Process domains were champions and leadership, with need to diversify championship among leaders. Relevant Intervention Characteristics included relative advantage and complexity, with need for personnel wellness initiatives and increased engagement with the community as service designers. Four recommendations included development of shared communication systems, personnel wellness campaigns, routine evaluations to inform practices, and culturally responsive care initiatives.

**Conclusion:**

Modifiable TIC determinants were identified, and a community-created implementation plan was developed to guide adoption. Future research will focus on city-wide implementation and strengthening pre-implementation research in other settings.

## Background

Psychological trauma disproportionately affects people with HIV (PWH) compared with the general population.^1–4^ Trauma refers to the harmful physical, psychological, social, or spiritual effects from exposure to adverse events (e.g., car accident, domestic violence, racism).^5–6^ Among PWH, these effects are associated with deleterious health outcomes, including accelerated immune system suppression and HIV disease progression, reduced medication and appointment adherence, and unsuppressed viremia.^1–2,4,7–8^ Associated psychiatric risks include substance use disorders, major depression, and Post-Traumatic Stress Disorder (PTSD), with a PTSD prevalence rate up to 74% among trauma-exposed PWH.^1,9^ Together, the synergistic effects of HIV and trauma constitute a syndemic^3,10^ and pose major barriers to the United States (U.S.) Ending the HIV Epidemic (EHE) goals.^11^

Consequent to the HIV-trauma syndemic, HIV care providers are repeatedly exposed to trauma as an occupational hazard.^12–13^ This increased trauma exposure among providers can negatively impact professional quality of life (PQoL);^12–14^ by reducing compassion satisfaction and leading to vicarious trauma.^15–16^ Over time, these exposures contribute to chronic exhaustion, reduced empathy, hypervigilance, minimization, a sense of persecution, guilt, anger/cynicism, addictions, and feelings of hopelessness.^13,16^ At least two-thirds of HIV care providers nationally experience burnout, including depersonalization, apathy, and disengagement from patients.^12,17^ These effects, in turn, are associated with reductions in patient satisfaction and provider trust, appointment non-adherence, and potential re-traumatization of PWH and their care providers.^18–20^

Trauma-informed care (TIC) is an evidence-based approach for mitigating the effects of trauma throughout healthcare systems.^12,21^ In TIC systems, personnel are trained and provided institutional resources with which to recognize and respond to trauma while resisting re-traumatization.^6^ Typically, the training and resources foster six general TIC principles: Safety; Trustworthiness and Transparency; Peer Support; Cross-Sector Collaboration and Mutuality; Empowerment, Voice, and Choice; and Cultural, Historical, and Gender Issues.^6,18^ TIC interventions are associated with decreased trauma effects, increased resilience, and improved HIV outcomes among PWH.^12,22–23^

Knowledge gaps remain around TIC implementation. First, little is known about effective implementation strategies. Although community engagement approaches like Community-Based Participatory Research (CBPR)^24^ align with TIC principles (e.g., collaboration and mutuality; empowerment, voice, and choice),^25^ limited literature depicts CBPR as a TIC implementation strategy. Second, more concrete operationalization of the TIC principle Cultural, Historical, and Gender Issues is required to actionize this principle. Cultural Responsiveness might be an appropriate operationalization of this principle^5,26–27^ and refers to how providers approach consumers with an understanding that individuals from different backgrounds experience and respond to trauma differently, and these differences can impact behaviors and relationships.^5^ However, no studies have explored how cultural responsiveness explicitly relates to TIC. Finally, few studies document TIC implementation in Southern community-based HIV Organizations (CBOs)^28^ or across city-wide HIV care networks. To address these gaps, we applied a CBPR approach to conduct pre-implementation stage research to develop a data-driven plan for implementing TIC across a city-wide HIV care network in the Southern U.S.

## Methods

### Study Setting

The Southern U.S. accounts for more than half of all new national HIV infections.^29^ In Nashville, Tennessee (TN), HIV rates are almost double that of the nation,^30^ and Black men who have sex with men (MSM) carry a disproportionate disease burden.^31^ A recent study shows PTSD rates are high (51%) among sampled PWH in the area.^22^ However, a local community-informed trauma intervention was associated with improvements in retention in medical care and viral suppression among trauma-affected PWH.^22^

### Study Participants

As part of the city-wide EHE initiative, overseen by the metropolitan mayor’s office, a TIC/ cultural humility workgroup representing five institutions was formed. Based on a pre-survey to assess priority topics and feasibility, workgroup members voted that implementation of TIC and cultural humility (i.e., as one category) was the greatest priority and most feasible. To design, implement, and evaluate city-wide implementation of culturally responsive trauma-informed HIV care (TIHC), a community-engaged researcher with experience implementing TIHC was asked to lead efforts.

Workgroup members then purposively sampled personnel from each of the city’s five highest-volume HIV care institutions, including two medical clinics, two community-based organizations, and one metropolitan health department. The targeted personnel represented a diverse range of perspectives, including leaders, direct care providers, support staff, and policymakers. Directors from these organizations informed personnel about the study and impending email invitations. Individuals agreeing to participate underwent an electronic, self-administered consents for key informant interviews. This study was approved by [REDACTED] IRB (protocol 21–05-1091) and [REDACTED] (protocol 2021004) under 45 CFR 46.110 (F) Category (7).

### Procedures

**Figure 1.**
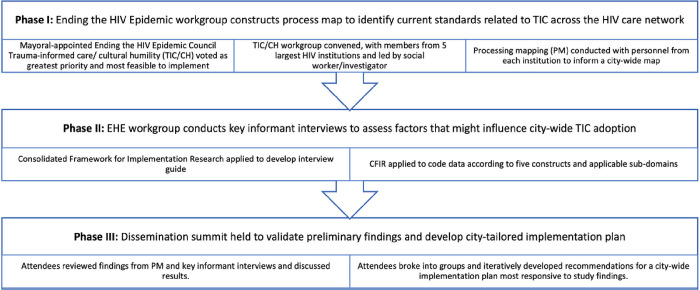
Pre-implementation stage research phases and activities guiding co-production of culturally responsive trauma-informed care across the HIV care network in Nashville, Tennessee

This study used a multi-method approach to explore standard care, perceived implementation determinants, and develop a data-driven implementation plan, involving three phases (See Fig. 1): I) process mapping (PM)^22,32–33^ II) key informant interviews, and III) a dissemination summit. Theoretical approaches were followed for conducting pre-implementation stage research^34^ and reporting qualitative findings (i.e., Consolidated criteria for reporting qualitative research).^35^

For Phase I, personnel convened between July 2021 and November 2022 to collaboratively construct PMs illustrating institutional practices and services relevant to TIC. Most discussions were conducted in person, with others held virtually during COVID-19 safety protocols. Attendees were provided presentations on the significance of TIC and HIV, asked to identify any current trauma screenings, assessments, services, and policies related to cultural responsiveness (e.g., systematic efforts towards diversity, equity, and inclusion), and interest in participating in TIC adoption. Groups met an average of four times to discuss map refinements. Consensus meetings were held with each institution to finalize individual maps and discuss forthcoming key informant interviews. Workgroup members synthesized all maps into one city map.

During Phase II activities, one-hour key informant interviews were conducted individually and virtually by two research assistants (one female and one male, one with a Masters of Public Health, and one with a bachelor’s degree); both had previously received training on qualitative interviewing and had no previous relationships with interviewees. Participants were aware of the study’s principal investigator, who was known in the community as interested in and overseeing implementation of TIC into HIV care. Phase I PM results were used to update a pre-existing interview guide.^36^ The adaptations focused half of the content on cultural responsiveness. Interviews were recorded and transcribed verbatim. Using a pre-existing codebook,^36^ three female study team members (one with a PhD in Social Work and some pre-existing relationships with participants, and two with Masters degrees, and one with a pre-existing relationship with some participants) applied thematic content analysis deductively using the Consolidated Framework for Implementation Research (CFIR).^37^ Findings were organized by the most current CFIR (1.0) constructs, including *Process, Characteristics of individuals* and *Intervention*, and *Inner* and *Outer setting factors*. An updated codebook was created initially to include the new cultural responsiveness themes with the first 20 interviews and to determine if data saturation had been reached. Findings were organized into relevant CFIR constructs, with coding decisions and discussions about construct definitions iteratively developed until 100% agreement was reached.

Finally, during Phase III activities, an additional group of personnel were convened for 1.5 days for an internal dissemination summit to develop a plan for co-producing^38^ TIC implementation. Attendees included local HIV care personnel (some who had been engaged previously for member-checking and some who had not yet been engaged in research activities) and an external facilitator. The city-wide map was presented, with a summary of interview findings and quotes. Three groups of 5–6 people then were asked to make recommendations to share with the larger group. The goal was to reach consensus based on need, urgency, and feasibility. Data was captured by three key study personnel recording notes during the summit and presenting the findings back to the group so that the group could reach consensus on major themes through workgroup discussions.

## Results

### Phase I: Process Mapping

**Figure 2.**
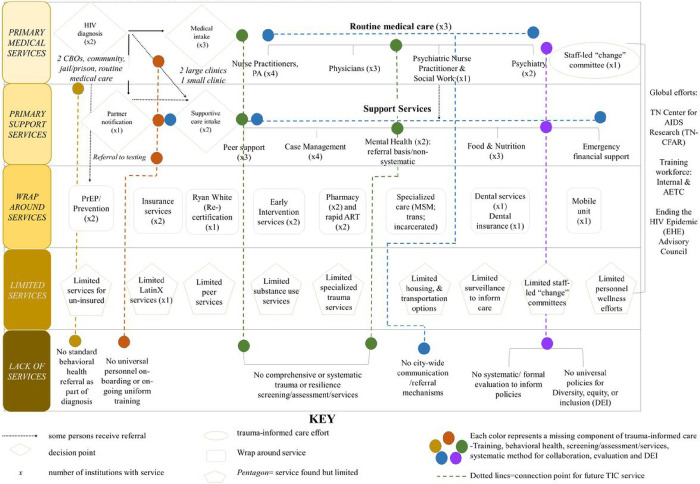
Process map depicting services and conditions relevant to trauma-informed care across a metro-wide network of HIV care in the Southern United States

Forty-eight personnel were engaged for PM discussions including 16 (33%) administrators/ leaders, 32 (67%) front-line/direct care providers/support staff, and 6 (13%) personnel with experience living with HIV. Findings from PM can be found in Fig. 2. Results suggest trauma screening and assessment differed by site, with one CBO and one clinic providing some level of trauma screening, assessment, and services but neither systematically or universally. The other CBO, clinic, and health department reportedly had no policies or procedures for regular trauma screenings and assessments and no trauma-specific interventions. Moreover, no institutions provided routine behavioral health screenings or referrals as a standard component universally for all new patients/clients, underscoring a critical gap in addressing the overall behavioral health of PWH. None of the institutions provided routine or standard training in trauma and trauma-informed care. Additionally, none of the institutions had current policies to explicitly promote diversity, equity, and inclusion (DEI) in the HIV care space. However, one clinic had an institution-wide policy (but nothing specific to the clinic); one CBO was working to integrate DEI into strategic planning, having begun early stages of antiracism efforts; and the health department had recently hired executive leadership to oversee an institution-wide DEI initiative. Additionally, while some institutions had existing personnel wellness initiatives, the need for improving HIV care provider emotional wellness and overall staff retention was emphasized.

### Phase II: Key informant interviews

Thirty-five personnel were interviewed, with ages ranging from 32 to 69 (median of 49). Eighteen identified as Black (51%) and 17 as White (49%); 26 as female (74.3%) and 9 as male (25.7%). Results from interviews are outlined below, by CFIR construct, and Table 1 provides example quotes for themes.

### Characteristics of Individuals

#### Knowledge, beliefs, self-efficacy, and prior experiences impacting personal characteristics.

Participants reported prioritizing patient-centeredness, trauma sensitivity, and attentiveness to fostering safe and supportive environments. Most felt prepared to respond to patient trauma due to knowledge acquired during years of relevant professional experience, education, and/or their own trauma experiences or membership within marginalized groups. All participants expressed support for TIC implementation and commitment to continuous learning to improve services and the mission to end the HIV epidemic through holistic approaches. Some participants felt under-prepared to deal with patient trauma due to lack of knowledge, feeling overwhelmed with personal trauma, the challenges of working in a state where policies are perceived to be oppressive towards minority populations, or the compounded stress of frontline work during a global pandemic.

“I could have been better prepared if I had deeper experience providing direct services myself [...] when you are in the trenches working with patients or clients, you learn so much more and grow. And so, that would’ve helped me, I think, understand some of the complexities that my staff are dealing with.” - Transcript 4: clinic leadership

### Inner setting

#### Culture

##### Norms, values, and basic assumptions in an environment related to its mission and purpose.

Organizational environments were described as inclusive, nonjudgmental, and patient-centered with compassionate staff who build trust through accountability, appropriate language/tone, protection of confidentiality, efforts to combat stigma/discrimination, and a trauma-sensitive service lens. Participants described a mission-driven culture where patient autonomy is promoted. Staff diversity was believed to be prioritized through hiring practices, with staff demographics often mirroring populations served. Some participants reported that representation is still a barrier, especially among clinicians, therapists, and leaders, with a perceived lack of value for diversity within upper management, which was experienced as re-traumatizing.

“Folks living with HIV [...], showing them that they can trust us [...] by just having the amazing staff that we have and building rapport and building relationships with people, and knowing that we have resources.” -Transcript 32: CBO provider

#### Networks and Communications

##### Nature and quality of internal social networks influencing work and patient care.

Overall, effective systems of internal communication were perceived to be in place for deescalating crises and supporting client relationships. Patient perspectives, needs, and feedback were informally captured during routine visits and shared in team meetings. Detailed intake assessments, advanced care plans, and mental health screenings were seen as important means of gathering and sharing patient information to provide the best care. Some participants reported fragmented teams and communication around crises. The degree of visibility of clients’ electronic medical records among providers garnered mixed perspectives, some advocating for greater visibility to avoid duplicative history-taking as potential re-traumatization, while others advocated for less visibility to protect confidentiality. Patients were nominally involved in organizational decision-making through community advisory boards (CABs) and ad hoc feedback surveys, which were cited as having unclear language, low client engagement, and poor representation among clients most difficult to reach. Similarly, CABs faced limitations such as scheduling conflicts, lack of client compensation, and insufficient policy and management support. Some noted feedback was often garnered from a few cherry-picked clients. The need for systematic and comprehensive consumer involvement and representative feedback was highlighted as necessary for a successful TIC program.

“Sometimes the peer team [is] overlooked in terms of what’s really happening with the clients. [...] clients may tell the case managers or even their therapists one thing and then we know an entirely different story, which usually we will tell the therapists if we refer them. [...] just because the clients feel more comfortable with us, they will really tell us a lot more, which would help with helping them with their issues...” -Transcript 29: CBO leadership

### Implementation Climate.

#### Tension for Change

##### Degree to which stakeholders perceive the status quo as intolerable and that change is necessary to improve care quality, client services, and staff wellness.

Difficulties in building client trust were connected to clients’ past negative experiences with providers, unaddressed mental illness, high staff turnover due to burnout, client disengagement related to provider apathy, racial tension between client and provider, provider perception that patients often do not progress, and a lack of client privacy with too many assigned providers. Participants reported that trauma screenings were not provided comprehensively, universally, or routinely, and that there is a need for better trauma screening tools and documentation of results. Most identified a need for more trauma support services to better address client trauma.

“They don’t trust people as a big umbrella. People may often come to us with a history of not being able to trust medical people. And so how do you establish that trust very quickly? I always tell a nurse, you get about 15 seconds when you walk in the room with that patient to establish a good relationship with them.” -Transcript 8: Clinic leadership

“So, us being well educated about the topic is pretty vital in helping people feel a little more comfortable. [...] but support system barriers and stigmas and self-stigma are huge.” -Transcript 32: CBO leadership

#### Compatibility

##### Alignment of intervention with personnel norms, values, perceived risks and needs, and fit with workflow and systems.

Participants reported that in addition to providing “excellent” clinical care, they work to destigmatize mental health services, foster trauma-sensitive spaces, and enrich interpersonal care by offering peer support groups among PWH. Participants unanimously recognized trauma as an issue affecting clients (e.g., trauma of HIV diagnosis/serostatus, childhood adversity, racial trauma) and acknowledged the need for TIC to optimize patient care. All supported the idea of working with a TIC coordinating program to improve TIC adoption. Many staff participants described current services as inherently trauma-informed but stated a need to prioritize staff wellness to improve retention of staff who work directly with traumatized patients while coping with their own trauma.

“[W]e could use trauma-informed care here to help the clients and measure client psychological wellbeing throughout the process. But also assist in supporting staff psychological wellbeing throughout the project. [...] it would be very valuable for clinics across the board.” -Transcript 10: clinic leadership

#### Learning Climate

##### Leadership transparency and collaboration in decision-making; personnel’s perceived value; safety of environment; time and space for reflective thinking and evaluation.

Participants reported several facilitators to address implicit bias in the workplace including open and honest communication in staff meetings, comfort with healthy disagreements, and a climate of continued learning through conversations and reflection. Past trauma trainings were described as culturally inclusive and well-explained in comfortable environments, which were seen as facilitative of meaningful discussions that helped providers feel more confident at work. Having supportive, available supervisors who value discussion and feedback and are familiar with staff responsibilities were noted as ways for personnel to feel heard and valued. However, some participants cited leadership issues in engaging staff, including a lack of follow through on suggestions, change-resistant leaders who are disconnected from frontline work, and a lack of leadership diversity. In some organizations, engagement in TIC was disincentivized as uncompensated work.

“One area where I struggle is when I see unconscious bias happening. They’re my coworker. So calling it out affects that relationship [...] the best way to enact change in that is not just having trainings, but also calling it out when you see it.” -Transcript 1: clinic provider

### Readiness for Implementation.

#### Leadership Engagement

##### Leadership commitment and involvement in TIC and prioritization of staff wellness.

Most participants could not elaborate on the extent of leadership’s support for TIC but noted there had been trainings, workshops, and/or discussion of TIC in meetings. Some noted that for a TIC program to be successful, leadership support and initiative was necessary, as was greater inclusion of community and personnel voice program design (i.e., versus leaders either designing programs without input or not being transparent about reasons or basis for changes). Participants in leadership roles, however, expressed support for TIC, along with barriers to implementation (e.g., lack of capacity/funding). Some leaders indicated their support of staff wellness, yet some personnel participants felt that their work-related trauma was not acknowledged by leadership and attributed high turnover rates to poor staff wellness. Some felt unheard and noted a need for more transparency in organizational decision-making.

“Like leadership, [...] they’re not really on the front, so they don’t really know what’s going on. [...] they’ll make a decision and you’re like, why did you make that decision that doesn’t work for us [...] because we see X amount of patients [...] I think sometimes leadership that aren’t directly here and see what goes on every day, they can make decisions [...] and trying to put that into practices isn’t that feasible because they don’t know the process.” -Transcript 12: health department provider

#### Available Resources

##### Resources dedicated to TIC.

Many participants credited their confidence in providing client care to a wealth of organizational resources and ongoing trainings. Some organizations provide a comprehensive “one-stop-shop” approach with several services available to clients within the same site. Participants viewed integrated onsite behavioral health services as a major benefit to staff when guiding patients through trauma and crises. Methods for promoting client safety included onsite services (e.g., security, mental health) and policies and procedures to promote respect and confidentiality. Some participants credited the work of committees, agency equity statements, and policies as assets for delivering culturally responsive services. Most participants identified capacity issues (e.g., short session times) as barriers to building trust, providing information, and empowering clients. Participants mentioned the need for onsite behavioral health, printed TIC materials, training to handle mental health crises for all staff, and increased staffing (especially Spanish speaking and therapists of color). Participants suggested TIC trainings should be reoccurring and incentivized (e.g., compensation or continuing education credits).

“One of the biggest challenges in working [as a provider] with our population is managing the effects of trauma that develop into behaviors [...] Sometimes 30 minutes isn’t enough to deal with some of those behaviors, but also get the information that I need to plus being therapeutic...” -Transcript 1: clinic provider

### Outer Setting

#### Patient Needs and Resources

##### Extent to which patient needs are assessed, known, and prioritized across systems.

A collective awareness existed about the need to screen and treat trauma, coupled with a recognition that mental health resources were lacking. Some viewed telehealth and online counseling as facilitators to meeting needs and increasing engagement, especially for those with transportation barriers. Barriers to providing care include clients’ unstable/unsafe home environments (e.g., intimate partner violence and challenges finding housing for clients with felonies), perceived judgement about medical adherence lapses, HIV stigma, and misinformation about HIV and sex education among family, friends, and PWH themselves.

“If a person doesn’t go to the doctor on a regular basis, for whatever reasons, lack of trust of the provider, we look at that as a failure, versus really trying to understand that. [...] but I think that a lot of people that have been lost care, if we unpack that, there’s more to just them not being able to have transportation to go to see their provider.” -Transcript 27: CBO leadership

#### Cosmopolitanism

##### Degree to which organizations are networked to support clients and TIC.

Many participants feel confident in providing services due to their knowledge of referral networks and relationships with community partners. However, some mentioned an overreliance on community partners, feeling ill-equipped to make referrals due to a lack of familiarity with external services, and the perception that institutions operate in silos from another. System-wide barriers to care include long wait times for external mental health support and insufficient HIV prevention resources. With federal regulations (i.e., HIPAA) complicating patient referrals and follow-up with community partners, many suggested a universal release of information. Collaboration among different organizations and across disciplines was seen as essential to the success of TIC adoption and EHE goals.

...[I]ndividualized training and cultural humility is an incredible step, but we’ve got to get our systems and processes smoothed out because we re-traumatize our patients when they have to re-disclose everything that they had just disclosed [...] We have to grow from TIC to better systems, and processes, and handoffs.

-Transcript 4: clinic leadership

### Process

#### Champions

##### Individuals bearing responsibility for influencing TIC implementation.

Some organizations have various committees serving as internal advocates for TIC and collaborating with external professionals. Participants discussed that more engagement from leadership, human resources, and equity teams is needed to promote cultural responsiveness. Many would like to see a TIC program created and uniformly applied across organizations as a mechanism for actionizing the goals of EHE. For city-wide TIC support, it was noted that the key to appealing to community engagement was to increase diversity among TIC champions.

“Rather than asking each institution to create their own TIC training, I would ask that you all create a training and you implement it the same throughout the institutions [...] if you’re asking us to do work having as much of it done [...] would be really helpful.” -Transcript 1: clinic provider

### Intervention Characteristics

#### Relative Advantage

##### Perceived impact of TIC on staff wellness and client services versus the status quo.

TIC and cultural humility trainings have helped participants respond to clients more confidently, but some TIC trainings have not adequately focused on vicarious and primary trauma among personnel. Some believe a city-wide effort to adopt TIC should be balanced with other priorities such as meeting clients’ basic needs. Some cautioned that the success of TIC programs rests on its relevance to the local community and needs appropriate, diverse, and representative developers, facilitators, and providers.

“You need to listen to the people when you’re developing programs [...] You have to make it unique to this metro community, and I think that we make a lot of mistakes where we think something works somewhere else, certain facilitators, certain people, and then we bring them and we apply them, and we think that’s going to take care of the problem.” -Transcript 27: CBO leadership

#### Complexity

##### Perceived difficulty of implementation (navigating systems for trauma service provision).

Participants anticipated experiencing challenges related to working within large, bureaucratic institutions, given the complexities of implementing a multi-level intervention. They saw hierarchies as barriers to collaborative decision-making, with financial and political considerations potentially being prioritized over patient-centered care. In some institutions, racism and inequity was described as part of leadership hiring practices and organizational structures of privilege. Some participants noted difficulties related to systems-level change, efforts to alter deep-seated beliefs, and stigma complicating client confrontation of trauma.

“We’re a clinic that operates within a larger corporate structure. [...] And so, there’re extreme limitations on what we can do, what authority patients have over this clinic [...] I have extreme limitations in my ability to initiate new programs.” -Transcript 4: clinic leadership

### Phase III: Summit implementation recommendations

Of the 17 individuals who attended the internal dissemination summit 10 (59%) were Black and 7 (41%) were White; 15 (88%) were female and 2 (12%) were male. Personnel included 5 (29%) direct care providers and 12 (71%) administrators, with approximately 3 (18%) persons either with lived experience with HIV or who have been affected by family members with lived experience. Summit participants discussed findings from the key informant interviews and made the following four overarching recommendations and relevant strategies. 1) Create a shared communication system to facilitate networking and referrals; strategies include convening routine cross-agency meetings for case conferencing, determining if there are dashboard-type models that exist or have been tested for implementation, and creating a clearing house for TIC training (i.e., to ensure all HIV care personnel in the system receive consistent training with attendance incentives). 2) Build a wellness campaign for HIV care personnel; strategies include providing on-site behavioral health support for employees or providing a mental health hotline for personnel (i.e., not relying solely on programs like employee assistance where scheduling, waiting, and co-pays can be barriers). 3) Implement routine evaluation to elicit and integrate patient and personnel feedback to design end-user systems of care; strategies include adding a step in the HIV care cascade explicitly for behavioral health, assessing organizational performance based around TIC metrics (e.g., the extent to which patients perceive their care environment to be safe), consistently evaluating personnel PQoL, and developing evaluation methods for all HIV care organizations to use uniformly to facilitate network-wide evaluation. 4) Prioritize and instill humanistic communication to foster culturally responsive care culture; strategies include diversifying leadership and practitioners/therapists, screening for cultural sensitivity as part of hiring new personnel, applying strengths-based models for patient care, providing consistent staff trainings that include experiential activities to model brave and open dialogue.

## Discussion

A CBPR approach was applied to collaboratively conduct pre-implementation stage research to develop a community- and data-driven TIC implementation plan for a metro-wide HIV care network. Results from PM and key informant interviews indicate there are limited trauma assessments and services provided in the HIV care network, but there is tension for change and an implementation climate compatible with TIC. There was an observed need for scaling up trauma services, personnel trauma training and wellness initiatives, systematic efforts to integrate end-user preferences into services, diversify leadership championship, prioritize and foster cultural responsiveness, and coordinate city-wide efforts by leveraging resources.

Our findings contribute to the TIHC knowledge base in several novel ways. To our knowledge, this is the first study to qualitatively explore implementation determinants of a city-wide trauma-informed HIV care network. However, the observed need for greater collaboration across the network is similar to previous TIC literature in other settings^13,20^ and collectively underscore the importance of network-level and multi-disciplinarian implementation efforts. The recommendation to launch a city-wide coordinating center appears to be a novel strategy for leveraging resources to build a greater macro-level infrastructure for HIV care. We believe our cultural responsiveness findings also mark the first of its kind (i.e., in an HIV setting as part of TIC) and provide empirical support for diversifying TIC championship and broadening efforts to integrate humanistic communication. Finally, our efforts to include community members or end-users as co-producers of TIC implementation appear to be unique and provide methods for future investigators conducting trauma-informed research (i.e., research conducted through methods that actionize TIC principles).

Though trauma was realized as pervasive and trauma services were a marked priority, trauma screening, referrals and services, and training were limited. There was a noted cost of maintaining the status quo (i.e., not implementing TIC) in which the network as a whole appears to insufficiently attend to mental health. While 83% of sampled Ryan White clinics in the Southeastern U.S. report providing mental health care on-site,^21^ 75% of HIV-focused CBOs in the Southern U.S. report insufficient community mental health services, and only 43% report having routine procedures for patient trauma screening.^28^ Our findings appear to be consistent with national,^20^ regional,^28^ and local findings, with the latter findings also indicating the need to improve the quality of mental health services in the catchment area.^31^ Moreover, consistent with TIC literature,^21,28,36,39^ we observed significant interest in TIC adoption within a workforce who have received little TIC training. These results underscore the need for carrying out the summit recommendation to create a shared communication system to facilitate networking, referrals, and training.

The noted need for greater staff wellness initiatives is also consistent with the TIC literature^21,39^ and appears to be a modifiable component of PQoL.^40–41^ While one-third of HIV care personnel report high levels of burnout,^21^ only one-third of CBOs address personnel vicarious trauma.^28^ This represents a critical omission given the trauma burden experienced by PWH, and the documented likelihood for vicarious trauma among HIV care personnel.^42^ While wellness campaigns excluding mental health have not improved clinical outcomes for staff,^43–44^ those including mental health support have been associated with reductions in stress, anxiety, and depression and improvements in well-being.^41^ Further, several strategies for improving personnel mental health, have been found to be effective in various settings.^40^ Hence, improving personnel mental wellness was found to be important according to personnel in the HIV care network, a critical aspect of wellness campaigns generally, and an effort supported by empirical findings.

The observed need for systematic efforts to engage community members or end-users as collaborative designers of services and policies is similar to that observed in other Southern CBO spaces.^28^ A recent quantitative study in the region showed a high rate of personnel reported working in systems in which patients are invited to share their thoughts, ideas, and experiences;^39^ our findings suggest future TIC research should assess whether feedback is systematic or informal, and whether feedback results are utilized to improve services. Overall, the limited systematic efforts to elicit and integrate patient experiences present a barrier to providing patient-centered care. A paucity of research focuses on how to best engage patients and other community members in HIV research,^45–46^ and future research should explore methods for increasing community-engagement as part of HIV research.

Empirical findings showing cultural responsiveness as a critical part of TIHC, mark the first of its kind. However, strategies raised during the summit (e.g., diversifying leadership and practitioners, screening during hiring procedures, providing consistent personnel trainings) are similar to many of those presented in recent literature on the role of antiracism in TIC.^47^ Further, the applied participatory approach and analysis of network-wide resources and strengths represents a strong mechanism for promoting culturally responsive research, given the raised sentiment that the success of a single institution undergoing cultural responsiveness initiatives would be potentially tempered if the whole metro system did not fully participate. This finding aligns with a conceptual article depicting the importance of cultural responsiveness as part of a city-wide effort to implement TIC within public health.^5^

This study has limitations. First, we employed purposive sampling that could have led to biases (e.g., perceptions about readiness for TIC). However, recruitment strategies were designed in unison with workgroup members and institutional leadership, which enhanced the collaborative nature of the approach. Second, while some thematic differences existed between organizations, this paper concentrates more broadly on city-wide themes due to the interdependent nature of shared clients and resources. Thus, some unique features of individual institutions may not appear thoroughly here. Finally, our findings cannot be generalized outside of the current sample because metro areas across the U.S. may face different barriers to TIC implementation. However, our theoretically-driven process, underpinned by implementation science and participatory methods, could be replicated by other TIC champions seeking to develop community- and data- driven implementation in large networks.

## Conclusions

Participatory methods were employed to develop a community-created and data-driven implementation plan to guide culturally responsive TIC throughout a network of HIV care in Nashville, TN. Current TIC standards were explored, as were personnel-perceived determinants of TIC implementation. A dissemination summit was convened to validate findings and generate a collaborative plan for future implementation. Findings contribute meaningfully to the growing body of TIC-HIV knowledge by showing the need to scale up trauma trainings, services, personnel wellness initiatives, and cultural responsiveness. Moreover, results suggest high levels of need for and compatibility with TIC across the Southern HIV care network. Future research will focus on maintaining community engagement to collectively guide TIC implementation. Researchers interested in co-produced TIC research may consider following similar methods to promote collaborative efforts aligned with TIC principles.

## Figures and Tables

**Table 1:** Perceived trauma-informed care implementation determinants throughout metropolitan Nashville

Domains & Constructs	Relevance to TIC	Example Quote
**CHARACTERISTICS OF INDIVIDUALS** Knowledge, beliefs, self-efficacy, and prior experiences impacting personal characteristics	Difficulty confronting bias and TIC resistance	“People from a marginalized group assume that they wouldn’t have implicit bias against another marginalized group ...to me is the biggest [barrier] with staff to get them to look at their own implicit biases.” -Transcript 27: CBO leadership
**INNER SETTING** *Culture:* Norms, values, basic assumptions in environment related to mission and purpose.	Culture of respect	“Stigma is a part of a lot of the conversations that we have on a regular basis [to] establish a culture of respect for our patients and acceptance of our patients...We’re not here to change anybody. We’re here to help them live the best life that they possibly can live.” -Transcript 4: clinic leadership
	System of retraumatization	“[P]eople are afraid of speaking up about injustices or ways that we’re re-traumatizing staff because they are experiencing it from the very people in leadership and administration... promotion of diversity never seems to be for leadership positions... the issue with white privilege and systemic racism is that the people in power to change that don’t want to give up their positions.” -Transcript 1: clinic provider
*Networks and Communications:* Nature and quality of internal social networks influencing work and patient care via decisionmaking.	Need for patient involvement in decision-making	“I think when you just start to design programs without any insight from those that are actually affected, you just lose that patient experience... because you’re not really addressing any of the stigma. You’re not addressing any of the patient’s lived experiences in your building programs...” -Transcript 14: health department leadership
**Implementation Climate** *Tension for Change:* Degree to which stakeholders perceive the status quo as intolerable and that change is necessary to improve care quality, client services, and staff wellness.	Stigma around psychological health	“Many of our clients...live day to day... just addressing...shelter, food, water, those things, and psychological and mental health needs tend to get put on the back burner a lot, but we know if those things don’t get addressed, everything can crumble...but a big barrier is so much stigma surrounding psychological health and wellbeing.” -Transcript 32: CBO provider
*Compatibility:* Alignment of intervention with personnel norms, values, perceived risks and needs, and fit with workflow and systems.	Client trauma is pervasive	“Every client who comes in has some sort of trauma, even if that trauma is just being diagnosed with HIV...” -Transcript 32: CBO provider
	Strong need to resist retraumatization	“[Patients] have a tendency to relive their trauma...It will overwhelm them sometimes and cause them to make not the best choices when they’re in a pressure situation. I think that given the correct guidance and tools, they would make better decisions...and try to make sure that if anything happens like that...they’re not triggered into making bad decisions again.” -Transcript 25 clinic provider
*Learning Climate:* Leadership transparency and collaboration in decision-making; personnel’s perceived value; safety of environment; time and space for reflective thinking and evaluation	Importance of transparency	“Consistency and transparency, willing to just be open about what’s actually going on in the agency and creating a space that we can provide feedback and that be taken in, but I think a lot of times they are reluctant to share certain information ... And so we don’t know what’s going on."
	Actions hinge on staff’s ability to voice opinions	“...you feel like, if you voice something, you feel like you’re going to definitely offend someone, so nobody really does anything, it never gets addressed.” -Transcript 35: CBO provider
**Readiness for Implementation** *Leadership Engagement:*Leadership commitment/involvement in TIC and prioritization of staff wellness	Need for stronger leadership engagement Compensating TIC engagement	“[Leaders] have to truly understand that if they don’t do this work, how it’s going to affect not only the constituents, and the clients, and patients that they serve, it’s also going to affect their personnel and their institution.” -Transcript 27: CBO leadership “Doing it [TIC committees] during clinical hours, so not expecting people to work for free during their afterhours.” -Transcript 1: clinic provider
*Available Resources:* Resources dedicated to TIC: funding, training, space/time, personnel, screening/assessment tools, programs/services/policies	Onsite Behavioral Health as an asset Turnover damages client trust Capacity barrier for TIC trainings	“Having behavioral health integrated is also a way of acknowledging and addressing stigma, having all these services under one roof is an intentional way of reducing stigma.” -Transcript 1: clinic provider “Turnover. That’s the worst thing that I think breaks and kills trust is when you start to open up to a provider or to a helper, and then they leave, and That’s been a huge barrier for us.” - Transcript 27: CBO leadership “Training for TIC is not a one and done process... it’s a continuous process of reevaluating and retraining. So, we currently don’t have a system where every six months we’re checking in ...and making sure that everyone has the same access to the same information.” Transcript 1: clinic provider
**OUTER SETTING** *Patient Needs & Resources:*Extent to which patient needs, and barriers and facilitators to meeting those needs, are accurately assessed, known, and prioritized across systems	Mental health care is a conduit for all care	“...substance abuse [...] pretty much takes over someone’s life. So if they’re not getting the help with substance use or mental health, then they’re probably not worried about taking their medications.” -Transcript 31: CBO provider
*Cosmopolitanism:* Degree to which care organizations are networked to meet client needs/support TIC	Need for city-wide collaboration	“[We need] a better conversation amongst the players in [the city]... we’ve got to get our systems and processes smoothed out, because we re-traumatize our patients...we have to grow from TIC to better systems, and processes, and handoffs.” -Transcript 4: clinic leadership
	Work guided by EHE goal	“If the goal is to end the epidemic, if it’s a part of the epidemic end points, then I would say primary care offices need to be engaged as much as HIV service organizations.” -Transcript 9: clinic provider
**PROCESS** *Champions:* Individuals appointed or volunteered to bear responsibility for or who influence TIC implementation	Recommendations for TIC roll out	“Really diversifying the ambassadors and the champions for trauma-informed care is...critical moving forward.” -Transcript 27: CBO leadership
	Efforts should be tailored to the metro system	“You need to listen to the people when you’re developing programs...make unique to this Metro community...we make a lot of mistakes where we think something works somewhere else... we apply them, and we think That’s going to take care of the problem. That’s been our biggest downfall.” -Transcript 27: CBO leadership
**INTERVENTION CHARACTERISTICS** *Relative Advantage:* Perceived impact of TIC on staff wellness and client services versus status quo	Need to further support both patients and personnel with trauma	“the underestimation of what a person has gone through is one of the biggest barriers that we face for being able to take care of patients.” - Transcript 5: clinic support staff "[Prior training] focused primarily on clients, and I don’t think that’s very beneficial when we have a lot of people who are of the community and also just experience trauma ourselves, and we are like reliving trauma everyday working with our clients.” -Transcript 35: CBO provider
*Complexity:* Perceived difficulty of implementation (navigating systems for trauma service provision)	Working within larger systems	“In a perfect world, we would give patients a voice at the table [and] decision-making authority... We’re a clinic that operates within a larger corporate structure...there’re extreme limitations on what we can do ...in my ability to initiate new programs.” -Transcript 4: clinic leadership
	General resistance to change	“Change is tricky and hard for a lot of people, and it’s really easy to just continue doing things the same way ...one of the biggest barriers is buy-in.” -Transcript 32: CBO provider
	Barriers to culturally responsive TIC	“The white privilege, the power and control of folks that don’t understand their own privilege and acknowledgement are ... huge barriers, and I mean, all from funders, to board, to leaders, to institutions where you refer clients, where clients receive services, it’s all the white privilege system.” -Transcript 27: CBO leadership

**Note:** Domains and constructs are from the Consolidate Framework for Implementation Research (Damschroder et al., 2009), with a specific focus on those presenting in the Implementation Research Logic Model (Smith, Li, Rafferty, 2020). “Relevance to TIC” column is based on authors’ interpretation of how specific implementation determinants relate to TIC as defined by the Substance Abuse and Mental Health Service Administration Treatment Improvement Protocol. 57.

## Data Availability

Data sharing is available for this manuscript.
